# There’s plenty of light at the bottom: statistics of photon penetration depth in random media

**DOI:** 10.1038/srep27057

**Published:** 2016-06-03

**Authors:** Fabrizio Martelli, Tiziano Binzoni, Antonio Pifferi, Lorenzo Spinelli, Andrea Farina, Alessandro Torricelli

**Affiliations:** 1 Università degli Studi di Firenze, Dipartimento di Fisica e Astronomia, Via G. Sansone 1, 50019 Sesto Fiorentino, Firenze, Italy; 2 Département de Neurosciences Fondamentales, University of Geneva, 1211 Geneva 4, Switzerland; 3 Département de l’Imagerie et des Sciences de l’Information Médicale, University Hospital, 1205 Geneva, Switzerland; 4 Dipartimento di Fisica, Politecnico di Milano, Milano, Italy, Piazza Leonardo da Vinci 32, 20133 Milano, Italy; 5 Istituto di Fotonica e Nanotecnologie, Consiglio Nazionale delle Ricerche, Piazza Leonardo da Vinci 32, 20133 Milano, Italy

## Abstract

We propose a comprehensive statistical approach describing the penetration depth of light in random media. The presented theory exploits the concept of probability density function *f*(*z*|*ρ*, *t*) for the maximum depth reached by the photons that are eventually re-emitted from the surface of the medium at distance *ρ* and time *t*. Analytical formulas for *f*, for the mean maximum depth 〈*z*_*max*_〉 and for the mean average depth 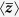 reached by the detected photons at the surface of a diffusive slab are derived within the framework of the diffusion approximation to the radiative transfer equation, both in the time domain and the continuous wave domain. Validation of the theory by means of comparisons with Monte Carlo simulations is also presented. The results are of interest for many research fields such as biomedical optics, advanced microscopy and disordered photonics.

In many research fields such as biomedical optics^1^, advanced microscopy^2^, or disordered photonics^3^ light is used as a tool to non-invasively extract useful information on what is below the surface of the related random media. The overall performance of this approach is strongly dependent on the exact physical description of the light-matter interaction, and this is more effectively provided within the framework of the radiative transport theory^4^. Usually, the medium is addressed in reflectance geometry, where light, injected and collected from the same side of its external surface, carries information on the medium optical properties encoded along photons random paths. For this reason, a key issue is often to increase as much as possible the depth reached by the migrating photons.

Depth information is crucial in brain functional imaging or in neuro-monitoring^5^,^6^,^7^, where a key challenge is the extraction of specific brain-cortex signals out of the superficial contamination generated, e.g., by scalp, skull and cerebrospinal fluid. In the breast spectroscopy the possibility to use hand held probes in reflectance geometry both for diagnostics or therapeutic monitoring^8^,^9^,^10^ raises issues on the real volume and depth reached by the measurements. Depth information is important also for other emerging applications such as cancer screening in thyroid or prostate^11^, or for non-clinical fields, such as internal quality assessment of agricultural produce^12^, non-destructive monitoring of wood materials^13^,^14^, or for pharmaceuticals and highly scattering plastics^15^. The rapidly growing field of sub-surface deep Raman spectroscopy^16^ is definitely interested in the depth profile of the measurement. On a different scale, optical microscopy demands the ability to reach large depths, in particular when dealing with intra-vital microscopy^17^. Similarly, the physics of optics in random media^3^,^18^,^19^, and the attempts to improve focusing through scattering media need to model the depth profile of re-emitted photons^20^,^21^,^22^,^23^.

The penetration depth of photons migrating in random media has been studied by several research groups^24^,^25^,^26^,^27^,^28^,^29^,^30^,^31^,^32^,^33^,^34^,^35^,^36^,^37^. The mean average depth 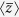 or the mean maximum depth 〈*z*_*max*_〉 reached by photons eventually detected at the surface of the medium at a distance *ρ* from the injection point are the main quantities investigated (also indicated as 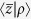 and 〈*z*_*max*_|*ρ*〉).

The pioneering work by Bonner *et al.*^24^ used a simple random walk model of light propagation to provide expressions for the continuous wave (CW) probability density of *z*_*max*_ for photons that emerge at a distance *ρ* from the injection point of light. Empirical formulas were proposed to estimate 〈*z*_*max*_〉 as a function of *ρ*, and limited Monte Carlo (MC) simulations were presented to validate the model. Interestingly the relationship 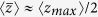 was presented as a result of the MC simulations, but no physical justification was included. In a later work^25^, 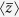 appeared to be proportional to *ρ*^1/2^, rather than *ρ*^2/3^ as previously presented^24^, but unfortunately no comments were made to justify the new results. This expression was confirmed by the exact enumeration method, but validation with MC results was not presented^26^,^27^.

In other papers the continuous time random walk model was used to derive analytical expressions for 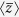 and 〈*z*_*max*_〉 in the approximation of large *ρ*^28^,^29^,^30^. Despite the absence of MC validation, the results supported the dependence of 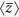 from *ρ*^1/2^. A similar result was proposed by Feng *et al.*^34^ within the framework of the diffusion theory in the strong absorption limit, while in the weak absorption limit a proportionality between 〈*z*_*max*_〉 and *ρ* was derived.

Zonios^37^ studied the penetration depth at short inter-optodes distances by means of MC simulations in the CW domain for a semi-infinite medium. For the total CW reflectance Zonios provided also an empirical analytical expression for 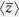 derived from MC simulations that shows to be valid even for large values of the reduced scattering coefficient, 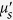, and the absorption coefficient, *μ*_*a*_, of the medium.

The first expression for 〈*z*_*max*_〉 in the time domain (TD) was presented by Weiss *et al.*^28^. Interestingly, in the TD 〈*z*_*max*_〉 was found to depend on *t*^1/2^, as previously experimentally shown by Patterson *et al.*^35^ and justified as a general behavior of diffusive (i.e. Brownian) particles. However, a dependence on *ρ* was also proposed, that was later theoretically and experimentally excluded by other works^38^,^39^,^40^. The approach used by Del Bianco *et al.*^40^ was based on the use of the cumulative probability to have *z*_*max*_ between *z* and *z* + *dz*, but the corresponding probability density function was not introduced. This characteristic limited the findings of this approach so that 〈*z*_*max*_〉 was obtained by an empirical relation and not by following a general theory.

Analytical expressions for the probability density of the residence time for trajectories constrained to reach position *ρ* at the time *t* in a semi-infinite medium (diffusion approximation) were proposed by Bicout *et al.*^31^,^32^, but validation with independent methods (e.g. MC) was not performed. Cui *et al.*^33^ experimentally showed that measured photons have longer mean path-lengths with larger variations if they reach greater depths. However, in these studies no results for 〈*z*_*max*_〉 or 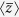 were reported.

An alternative way to address the penetration depth of light was proposed by Carp *et al.*^36^ that used two possible depth metrics for such purpose: a first one given by the depth at which the fluence rate falls to 1/*e* of the incident fluence rate, and the second one given by the depth at which 1/*e* of the laser radiation has been absorbed. The first metric focuses on the intensity distribution inside the medium, while the second one focuses on the absorption depth of the propagated light.

From the above observations, the lack of a general and precise formulation for the problem of assessing the average penetration depth of migrating photons becomes evident. Even with this huge amount of work, it remains extremely difficult to make a clear picture of the dependence of 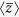 and 〈*z*_*max*_〉 from the physical parameters that characterize the medium (i.e. *μ*_*a*_ and 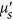) and from the parameters (e.g. *ρ* and *t*) that inherently depend on the type of measurements (i.e. CW or TD).

Moreover, the proposed theoretical expressions, the results of the numerical simulations or the findings of the experimental tests, appear to be generally fragmented and only partially validated by independent methods (e.g. by MC simulations). This is a limitation to the actual practical use of the previous developed theories.

The aim of this work is to give a comprehensive analytical description of the statistics of the penetration depth of light in a turbid medium within the framework of the Radiative Transfer Equation (RTE) for both TD and CW domain. The following points are considered: 1) a general method for the derivation of the probability density function that the photons emerging from an infinite slab have a *z*_*max*_ between *z* and *z* + *dz* is presented; 2) the results for 〈*z*_*max*_〉 in the diffusion approximation (DA) for the CW and the TD are proposed and systematically validated by “gold standard” MC simulations; 3) from the mean maximal depth 〈*z*_*max*_〉 the mean average depth 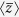 is also obtained.

Explicit calculations are thus obtained analytically with the DA, and numerically by means of MC simulations. Details on the MC calculations are reported in section Methods. In section Discussion we finally express some general comments on the obtained results and on their impact in the scientific community.

## Theory

### Statistics of photon penetration depth inside an infinite slab

**Figure 1:**
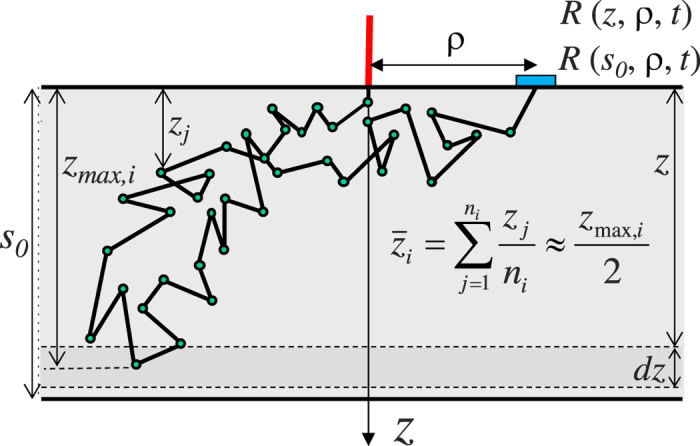
Schematic for a 2D projection of a detected photon trajectory from a diffusive slab: *z*_*j*_ is the value of the *z* coordinate (depth) when photon experiences a scattering event along the detected trajectory *i*. *n*_*i*_ is the number of scattering events in the detected trajectory *i*.

The theory presented in this section holds in general within the framework of the RTE. Let’s consider an infinite slab of finite thickness *s*_0_, reduced scattering coefficient 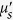, absorption coefficient *μ*_*a*_, where a pencil beam impinges perpendicularly to the entrance surface of the slab. Our aim is to define the probability density function that the emerging photons from the medium have a *z*_*max*_ between *z* and *z* + *dz*. This function can be derived comparing the signal detected on two slabs with thicknesses *z* and *z* + *dz*, respectively and located inside the original slab *s*_0_ (see Fig. 1).

The difference in signal between the two cases is indeed due to photons that have trespassed *z* but not *z* + *dz*. For a slab of thickness *s*_0_, the fraction of photons exiting the medium at *ρ* and *t* with the maximum penetration depth *z*_*max*_ between *z* and *z* + *dz* (with the *z* axis along the incident pencil beam) is given by the ratio


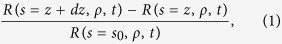


with *R*(*s*, *ρ*, *t*) the TD reflectance at *ρ* from a slab of thickness *s*. Thus, we can define the corresponding probability density function, *f*(*z*|*ρ*, *t*), for the slab of thickness *s*_0_ in the range 0 ≤ *z* ≤ *s*_0_ as


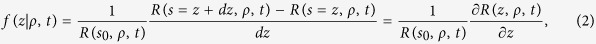


where *f*(*z*|*ρ*, *t*)*dz* is the probability that the emerging photons from the slab at *ρ* and *t* had a *z*_*max*_ between *z* and *z* + *dz*. It is worth noting that *f*(*z*|*ρ*, *t*) depends on 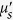 and that this dependence will always be present in all subsequently derived formulas. Instead, thanks to the general properties of the RTE^41^,^42^, for a homogeneous medium the dependence on *μ*_*a*_ vanishes.

The same definition can be given for the total TD reflectance, 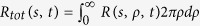, and we obtain


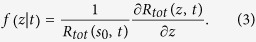


We note that *f*(*z*|*t*) can also be obtained as


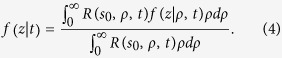


The probability density function *f*(*z*|*ρ*, *t*) provides a detailed description of the distribution of *z*_*max*_ for the photons emerging from the medium. The above definition of *f*(*z*|*ρ*, *t*) is general and can be used without any restriction within the framework of the RTE. Thus, *f*(*z*|*ρ*, *t*) can be practically calculated for homogeneous and inhomogeneous geometries with analytical solutions of the Diffusion Equation (DE) or of the RTE for the reflectance *R*(*s*, *ρ*, *t*).

Making use of *f*(*z*|*ρ*, *t*) it is then possible to calculate 〈*z*_*max*_|*ρ*, *t*〉, the mean value of the maximum penetration depth *z*_*max*_ of the photons emerging from the medium at distance *ρ* and at time *t* as:


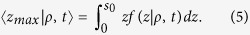


In analogous way we can also define 〈*z*_*max*_|*t*〉 as


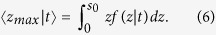


Thanks to the RTE properties for homogeneous media 〈*z*_*max*_|*ρ*, *t*〉 and 〈*z*_*max*_|*t*〉 are also independent of *μ*_*a*_, while they still depend on 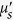. From now onward we implicitly assume that all the TD formulae are independent of *μ*_*a*_.

Similarly to the TD we can act in the CW domain and we can define a probability density function, *f*(*z*|*ρ*),


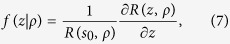


with 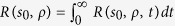 reflectance for the CW source. We note that *f*(*z*|*ρ*) can also be obtained by the average of *f*(*z*|*ρ*, *t*) over *t* using the function *R*(*s*_0_, *ρ*, *t*) as weight factor, i.e.,


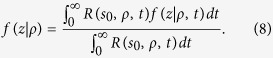


For the CW total received light, 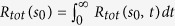, we also have *f*(*z*) defined as


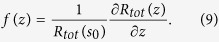


We can express the mean maximum penetration depth for CW light, 〈*z*_*max*_|*ρ*〉, as


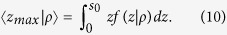


Similarly, by using the total CW reflectance, *R*_*tot*_(*z*), we can define 〈*z*_*max*_〉 as


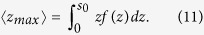


We note that, in contrast to the TD, in the CW domain both *f*(*z*|*ρ*), *f*(*z*), 〈*z*_*max*_|*ρ*〉 and 〈*z*_*max*_〉 depend also on *μ*_*a*_.

All the above definitions can be extended to a semi-infinite medium by considering a slab with a sufficiently large thickness. This is an important extreme case that has been extensively studied in the literature. Therefore, in the section Results we will present some data for the semi-infinite medium and the slab.

The above theoretical approach is completely consistent within the framework of the RTE when Fresnel reflections do not occur at the external boundaries of the slab. Conversely, when there is a refractive index mismatch between the medium and the external space (*n*_*rel*_ ≠ 1), we need to provide a correction to the above theory. In the definition of *f*(*z*|*ρ*, *t*) we need to consider that for the slab of thickness *z*, having its upper surface coincident with that of the slab *s*_0_ and its lower surface *internal* to it, no Fresnel reflections occur at its lower boundary. Thus in this case the reflectance *R*(*s* = *z*, *ρ*, *t*) in Eq. (2) has to be replaced by a reflectance term *R*′(*s* = *z*, *ρ*, *t*) free of Fresnel reflections at depth *z*. The comparisons with the results of MC simulations show that this correction leads to an accurate calculation of *f*(*z*|*ρ*, *t*) also when *n*_*rel*_ ≠ 1.

### Expressions for *f* and 〈*z*_*max*_〉 for an infinite slab in the diffusion approximation

The DE provides a wide set of approximate analytical solutions of the RTE for various geometries^41^,^42^. We consider in this section the analytical solutions of the DE for an infinite slab and for this geometry we calculate *f*_*DE*_(*z*|*ρ*, *t*), 〈*z*_*max*_|*ρ*, *t*〉_*DE*_, *f*_*DE*_(*z*|*ρ*), 〈*z*_*max*_|*ρ*〉_*DE*_, *f*_*DE*_(*z*) and 〈*z*_*max*_〉_*DE*_.

If we consider the analytical TD solution of the DE for an infinite homogeneous slab^41^,^42^, *R_DE_*(*s*, *ρ*, *t*), the dependence on *ρ* is only the multiplicative factor 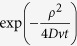
42, with 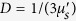 diffusion coefficient, and *v* speed of light in the medium. Thus, the dependence on *ρ* vanishes in *f*_*DE*_(*z*|*ρ*, *t*) and therefore also in 〈*z*_*max*_|*ρ*, *t*〉_*DE*_. We stress that a similar term appears also for other homogeneous geometries with an unbounded lateral dimension^42^. Then, when dealing with the DE solutions in homogeneous unbounded geometries, the above formulae will be simply denoted as *f*_*DE*_(*z*|*t*) and 〈*z*_*max*_|*t*〉_*DE*_. The above property implies that


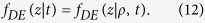


Thus, for the calculation of *f*_*DE*_(*z*|*ρ*, *t*) we can use any value of *ρ* or alternatively the total reflectance *R*_*totDE*_(*s*, *t*) instead of *R*_*DE*_(*s*, *ρ*, *t*). The practical consequence of this fact is beyond the diffusion approximation since, as it will be shown in section Results, it is practically valid for most of the time ranges of interest for applications. About the DE solutions for the slab we have verified that we obtain identical probability density functions when using *R*(*s*, *ρ*, *t*) and *R*_*tot*_(*s*, *t*). Then, the analytical expression of *f*(*z*|*t*) can be obtained by using the solution of the DE for the infinite slab presented in refs 41,42. By means of Eq. (4.27) in ref. 42 we express 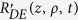 as a series expansion:


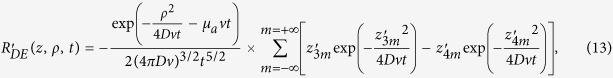


where


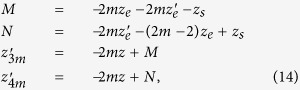


and by using Eq. (2) we have that *f*_*DE*_(*z*|*t*) is


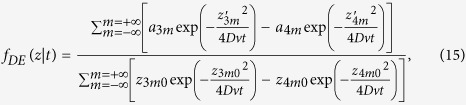


where


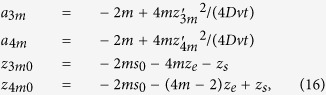


with 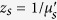 position of an isotropic source of unitary strength used to model an external pencil beam of unitary strength impinging onto the slab^41^,^42^, *z*_*e*_ = 2*AD* extrapolated distance obtained in accordance to the extrapolated boundary condition (*A* coefficient for the Fresnel reflections, see refs 41,42), and 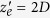 extrapolated distance in absence of Fresnel reflections. The above formula is indifferently valid when Fresnel reflections occur at the external boundary (*n*_*rel*_ ≠ 1) or in absence of reflections (*n*_*rel*_ = 1). For obtaining the above formula we have used the correction proposed in the previous section for the case *n*_*rel*_ ≠ 1.

Making use of Eq. (5), where we replace *f*(*z*|*ρ*, *t*) with *f*_*DE*_(*z*|*t*) (Eq. (15)), and after solving the integral in *z*, we obtain the analytical solution for 〈*z*_*max*_|*t*〉_*DE*_ that is


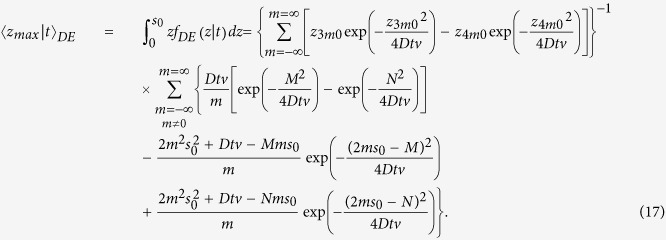


For the CW domain we act similarly to the TD starting from the analytical solution for the reflectance, 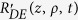



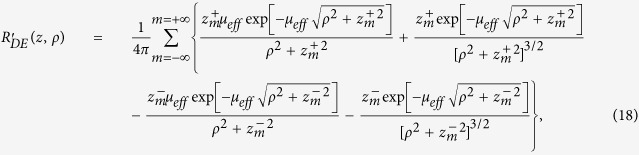


where


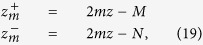


with 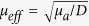 effective attenuation coefficient. For the studied slab of thickness *s*_0_ we have that


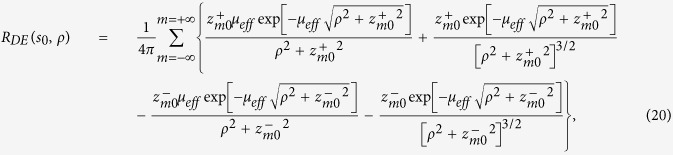


where


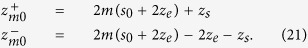


By using Eqs (7), (18) and (20) we have that *f*_*DE*_(*z*, *ρ*) is


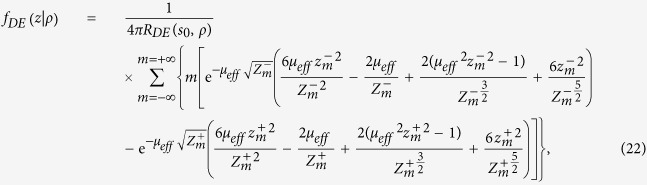


with


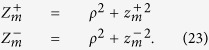


By using Eqs (10) and (22) we obtain 〈*z*_*max*_|*ρ*〉_*DE*_


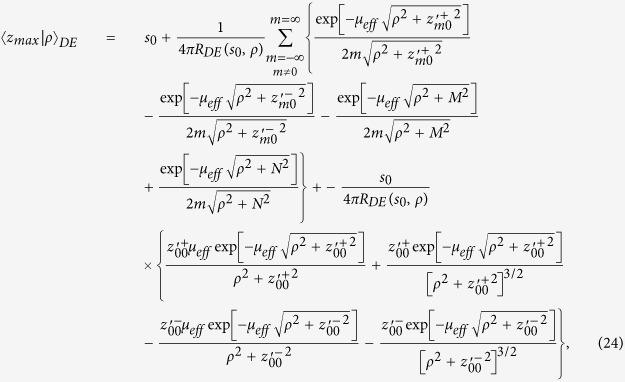


where


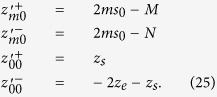


It is possible to verify that for a non-absorbing slab 
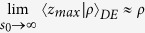
. Thus, for a non-absorbing semi-infinite medium we have a quite simplified calculation. We also note that, due to the difficulty to compute the exact limit of Eq. (24) for the special case *μ*_*a*_ = 0, this particular value can be more easily calculated by approximating *μ*_*a*_ to a very small value.

For the CW domain the same calculations can be also implemented for the total reflectance, that for the slab of thickness *z* can be written as


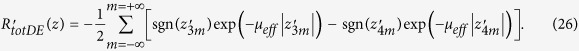


Making use of Eqs (9) and (26) we can derive the following analytical solutions for *f*_*DE*_(*z*):


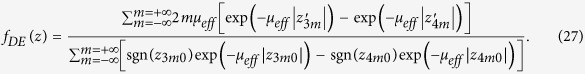


By using Eqs (11) and (27) we finally obtain 〈*z*_*max*_〉_*DE*_


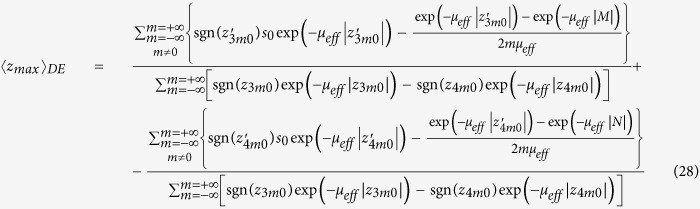


where


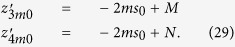


We note that for a non-absorbing slab Eq. (28) is undetermined. Thus, the special case *μ*_*a*_ = 0 must be calculated by approximating *μ*_*a*_ to a very small value.

At this point, we must stress that the normalization of the probability density functions *f*_*DE*_(*z*|*t*), *f*_*DE*_(*z*|*ρ*) and *f*_*DE*_(*z*) is subjected to the conditions *R*_*DE*_(0, *ρ*, *t*) = 0, *R*_*DE*_(0, *ρ*) = 0 and *R*_*totDE*_(0) = 0 (see Theory section), whose validity is expected for obvious physical reasons. Within the framework of the DA these conditions are usually well verified as shown in the following sections. However, we must warn that for high values of *μ*_*a*_ and small values of *s*_0_, when the DA is expected to fail, the above probability density functions may be affected by normalization deficiencies. In the Results section we will see that the proposed solutions are actually very stable and often hold also beyond the diffusive regime.

### Heuristic formula for the mean penetration depth 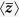 in a homogeneous medium

Equation (5) provides the mean value of the maximum penetration depth reached by photons exiting the medium at distance *ρ* and time *t*. It would be also interesting to know the mean value 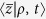 at which detected photons have undergone scattering events, i.e. the average value of the coordinates *z* of all the scattering events of the detected trajectories. As suggested by Bonner *et al.*^24^, an intuitive heuristic relation can be given as follow:


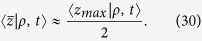


Similarly, for the CW domain we can express the mean value of the coordinates *z* of the photons exiting at *ρ* in the medium with absorption *μ*_*a*_ as


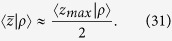


In fact, Eqs (30) and (31) can be heuristically justified by representing a single trajectory *i* in a chaotic diffusive regime of photon migration (see Fig. 1). Given the maximum penetration *z*_*max*,*i*_ of this trajectory we have roughly that


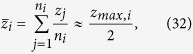


where *z*_*j*_ is the value of the *z* coordinate (depth) when photon experiences a scattering event along the detected trajectory *i*, and *n*_*i*_ is the number of scattering events in the detected trajectory *i*. In absence of reflections at the external boundary the relation is well verified since there are not physical reasons that may render preferable for photons to migrate in the superficial layers or in the deep part of the medium. However, when there is a refractive index mismatch, Fresnel reflections determine an increase of the probability to have photons in the superficial layer of the medium so that 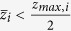. Equation (30), although approximated, it is well verified for trajectories with a high number of scattering events, while provides a poor description for detected light subjected to few scattering events inside the medium. For instance, photons detected after a single scattering event verify the condition 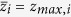 and the above relation clearly fails. We finally note that when the total reflectance from the medium is considered the same equivalent heuristic relations of Eqs (30) and (31) can be written between 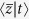 and 〈*z*_*max*_|*t*〉 and between 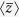 and 〈*z*_*max*_〉. It is interesting to note that Bonner *et al.*^24^ found the relation of Eq. (31) as a result of MC simulations, but no physical justification was included in their work. In the present contribution, it will be shown that Eqs (30) and (31) are actually valid within a diffusive regime of light propagation.

## Results

This section is dedicated to the presentation of the probability density functions and of penetration depths calculated in the previous sections, both in TD and CW domain, and derived from the DE. These results are validated with the outcome of MC simulations (see Methods section), used as a reference standard. First, we verify by means of MC simulations that the TD results are independent of *ρ*. This result allows us to use the total reflectance, *R*_*tot*_(*s*, *t*), in the MC calculations and drastically improves the convergence speed. Then, we compare the TD and CW probability density functions obtained with the DE and with MC simulations. Finally, we present the MC/DE comparisons for 〈*z*_*max*_|*t*〉, 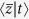, 〈*z*_*max*_|*ρ*〉, 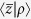, 〈*z*_*max*_〉 and 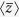.

### Independence of the TD results from the source-detector distance *ρ*

In the Theory section we have seen that, within the diffusion approximation, the TD expressions for both *f*(*z*|*ρ*, *t*) and 〈*z*_*max*_|*ρ*, *t*〉 for unbounded geometries are independent of the source-detector distance *ρ*. We first investigate if the validity of this property is also confirmed within the RTE by using MC simulations. If validated, this property allows to improve the efficiency of the MC method when studying the penetration depth. In this section we focus our study on the mean maximum penetration depth 〈*z*_*max*_|*ρ*, *t*〉.

**Figure 2:**
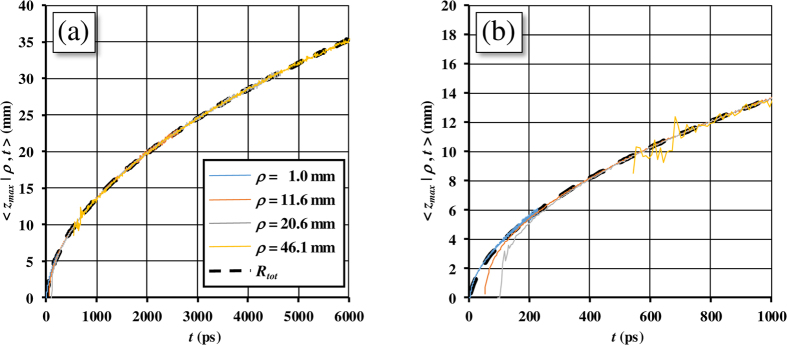
Mean maximum TD penetration depth, 〈*z*_*max*_|*ρ*, *t*〉, obtained with MC simulations plotted versus time for different *ρ*. A semi-infinite diffusive medium with *μ*_*a*_ = 0, 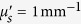 and *n*_int_ = *n*_ext_ = 1.4 has been considered. The result obtained for the total reflectance *R*_*tot*_ is also shown (black dashed line). In panel (**b**) it is shown a detail of panel (**a**) with an emphasis on the time range from 0 to 1000 ps.

Figure 2 shows 〈*z*_*max*_|*ρ*, *t*〉, obtained from MC simulations, for a semi-infinite medium as a function of the photon time of flight *t* for a large set of *ρ*. In the figure it is also shown 〈*z*_*max*_|*ρ*, *t*〉 obtained from the fast MC method exploiting *R*_*tot*_(*s*, *t*). It can be noticed that the differences between the two methods are very small except for the very early times (see Fig. 2b), corresponding to the rising edge of the temporal profile, and for which the amount of light involved in the detected reflectance is in any case negligible. This fact proves that the independence of the penetration depth from *ρ*, exactly true within the DE, is in practice valid also within the RTE, with the only exception of the very early times. For example, at *ρ* = 20.6 mm the agreement is very good for *t* > 300 ps, while at *ρ* = 11.6 mm it is already good at *t* > 200 ps. Thus, this result justifies the use of the total reflectance for calculating the functions *f*(*z*|*t*), 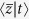 and 〈*z*_*max*_|*t*〉, and this is what has been done in the present contribution. Similar results are obtained when a slab of finite thickness is considered (data not shown).

### Comparisons MC/DE for *f*(*z*|*t*) and *f*(*z*|*ρ*)

Here we report some examples for the TD and CW probability density functions *f*(*z*|*t*) and *f*(*z*|*ρ*) obtained with MC simulations and the corresponding quantities *f*_*DE*_(*z*|*t*) and *f*_*DE*_(*z*|*ρ*) calculated with Eqs (15) and (22).

**Figure 3:**
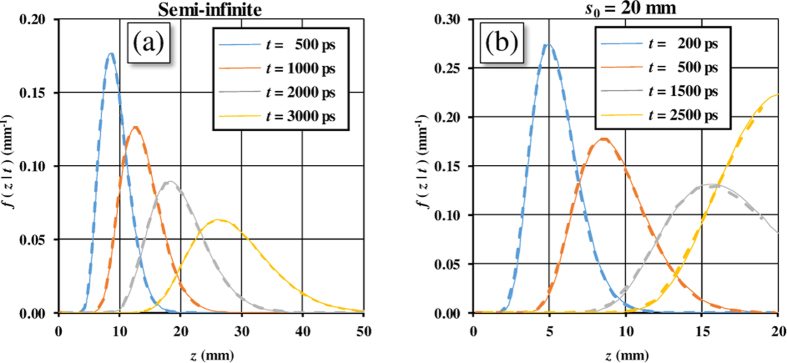
Probability density function *f*(*z*|*t*) versus the depth *z* for different times *t*, calculated exploiting both DE (solid lines) and MC (dashed lines). The figure pertains to a semi-infinite medium (a) and to a slab with *s*_0_ = 20 mm (b), with *μ*_*a*_ = 0, 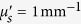 and *n*_int_ = *n*_ext_ = 1.4.

In Fig. 3a, the probability density functions *f* calculated exploiting both DE and MC are reported as a function of *t* for a semi-infinite diffuse medium with *μ*_*a*_ = 0, 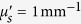 and *n*_int_ = *n*_ext_ = 1.4. As one can see, the two approaches are indistinguishable. Some differences are only visible at very early times (<100 ps, data not shown). Figure 3a shows how the probability distribution for the maximum penetration depth *z*_*max*_ moves towards deeper values of *z* by increasing the time, while the width of the curve becomes larger and larger. In Fig. 3b the results for a slab of 20 mm thickness are reported, confirming the agreement between DE and MC and the general behavior for the probability density function. The domain of definition of *f*(*z*|*t*) is in this case the interval [0, 20] mm, set by the slab thickness *s*_0_.

**Figure 4:**
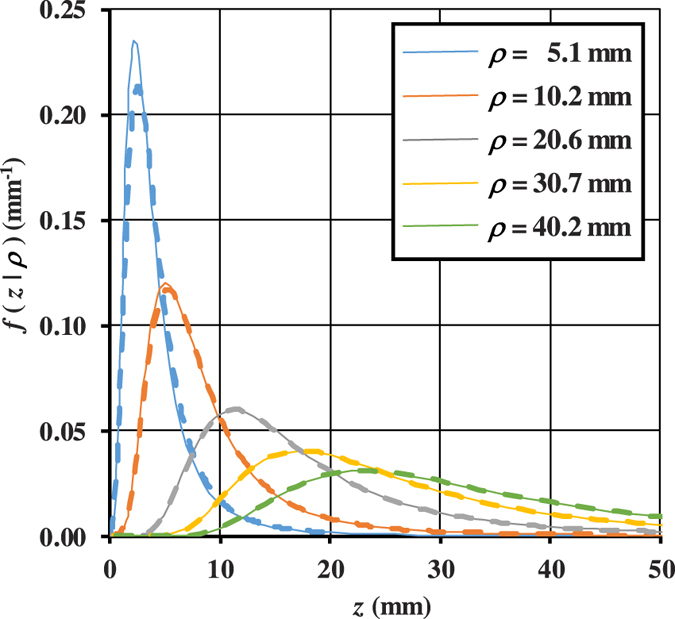
Probability density function *f*(*z*|*ρ*) versus *z* for different *ρ*, calculated exploiting both DE (solid lines) and MC (dashed lines). A semi-infinite diffusive medium with *μ*_*a*_ = 0, 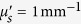 and *n*_int_ = *n*_ext_ = 1.4 has been considered.

In the CW domain, the probability density function *f*(*z*|*ρ*) depends on 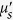, *ρ*, and *μ*_*a*_. The function *f*_*DE*_(*z*|*ρ*) is the integral of *f*_*DE*_(*z*|*t*) and *R*(*ρ*, *t*) (Eq. (8)). Thus, the validity of *f*_*DE*_(*z*|*ρ*) is implicitly guaranteed by the accuracy of *f*_*DE*_(*z*|*t*) and *R*(*ρ*, *t*) that have been validated elsewhere (Fig. 3, and ref. 42). For the sake of completeness, *f*_*DE*_(*z*|*ρ*) is here validated also by the MC. In Fig. 4 *f*(*z*|*ρ*) calculated from Eq. (22) is compared with the results of MC simulations for a non-absorbing semi-infinite medium and for different values of the source-detector separation *ρ*. The comparisons show that, except short *ρ* for which the DE does not hold, the agreement between analytical formulae and MC simulations is excellent. We note that in Fig. 4 *z*_*max*_ has a probability density function with the maximum peak placed at larger values of *z* as *ρ* increases, and that the curves become more and more asymmetric with a long tail for larger *ρ*. This behavior is emphasized by the absence of absorption.

Very similar results were obtained for the probability density functions when we assume a refractive mismatch with the external region (data not shown).

### Comparisons MC/DE for 〈*z*_*max*_|*t*〉, 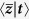 , 〈*z*_*max*_|*ρ*〉, 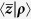 , 〈*z*_*max*_〉 and 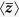


**Figure 5:**
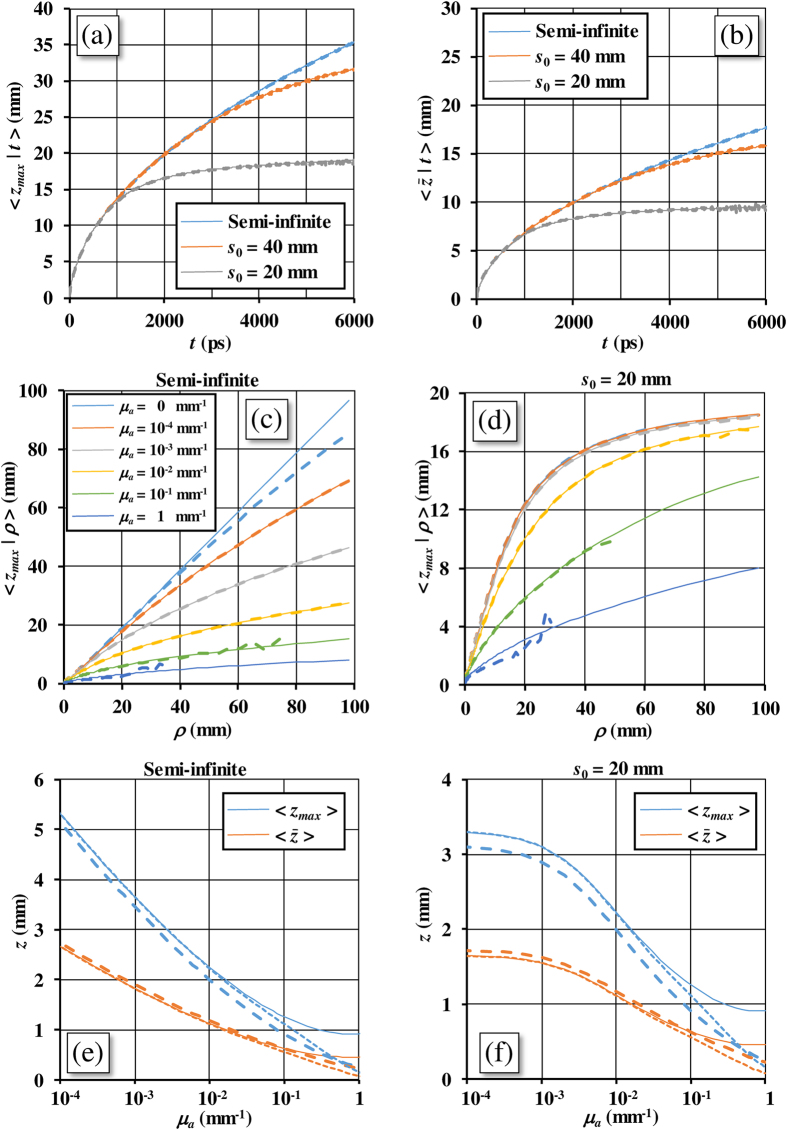
TD case: 〈*z*_*max*_|*t*〉 (**a**) and 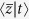 (**b**) versus time. CW domain case: (**c**,**d**) 〈*z*_*max*_|*ρ*〉 for different values of *μ*_*a*_; (**e**,**f**) 〈*z*_*max*_〉 and 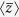 versus *μ*_*a*_. The quantities were calculated both with DE (solid lines) and MC (dashed lines). In (**e**,**f**) also results of the DE solutions obtained placing the source at 
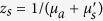
 are reported (dotted lines). Panels (**a**,**b**) pertain to a semi-infinite diffusive medium and to a slab with *s*_0_ = 20, 40 mm. Panels (**c**,**e**) pertain to a semi-infinite diffusive medium, while panels (**d**,**f**) pertain to a slab with *s*_0_ = 20 mm. For all the panels we have 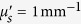 and *n*_int_ = *n*_ext_ = 1.4.

Figure 5a,b shows 〈*z*_*max*_|*t*〉 and 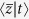 according to Eqs (17) and (30) using the DE. The results shown pertain to slabs of thicknesses 20, 40, and to a semi-infinite medium with 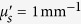, *n*_int_ = *n*_ext_ = 1.4. The same quantities calculated by means of MC simulations (see section Methods, Eqs (33) and (34)) are reported on the same plots for validation. The DE data are in excellent agreement with the MC results. Furthermore, 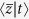 computed with Eq. (30) is also perfectly validated by the MC. Figure 5a shows that 〈*z*_*max*_|*t*〉 grows rapidly for early times and more slowly for late times. Five ns are needed to penetrate at 30 mm depth in the medium. We note that 〈*z*_*max*_|*t* → ∞〉 ≈ *s*_0_, while for a semi-infinite medium it increases indefinitely.

Figure 5c shows 〈*z*_*max*_|*ρ*〉 as a function of *ρ*, for a wide range of *μ*_*a*_ ∈ [0, 1] mm^−1^, from a semi-infinite medium. The results obtained with DE, i.e. Eq. (24), and MC are reported. For values of *μ*_*a*_ ∈ [10^−4^, 10^−1^] mm^−1^, typical to the absorption of biological tissues in the near infrared spectral region, the agreement between DE and MC is very good at any *ρ* ∈ [0, 100] mm. We note large differences only for the non absorbing medium and for *μ*_*a*_ = 1 mm^−1^. In the case *μ*_*a*_ = 0 the reasons of the differences at larger *ρ* can be ascribed to limitation of the the MC method that would require prohibitive values for the maximum length of each trajectory. In the simulations shown, although each trajectory has been actually followed up to a maximum length of 43 m, we still note that this length is not enough to correctly evaluate the detected signal. About the MC simulations in a non-absorbing semi-infinite medium we have thus a physical limit of the numerical procedure that cannot be correctly implemented when we consider large *ρ* values. This effect in Fig. 5c is limited to the data with *ρ* > 50 mm. Notwithstanding, the almost perfect agreement between MC and DE obtained for higher values of absorption guarantees that there are not physical reasons to justify a failure of Eq. (24) for *μ*_*a*_ = 0. On this ground, we have full confidence that the values of the DE obtained with Eq. (24) for *μ*_*a*_ = 0 and the semi-infinite medium (Fig. 5c) have the same accuracy observed for the other higher values of absorption so that the main limitations in this case only pertain to the MC. The figures also show that as *μ*_*a*_ increases, much larger distances have to be used to reach the same depth. For the highest absorptions *μ*_*a*_ = 0.1 and 1 mm^−1^ the noise of the MC data compromises a part of the MC results that are therefore not reported at large distances. We observe that for high *μ*_*a*_ values we still have a surprising good MC/DE agreement although we could expect a breakdown of the DA in this region. In Fig. 5d the same results for 〈*z*_*max*_|*ρ*〉 have been shown for a slab with *s*_0_ = 20 mm for which we observe an overall excellent agreement between MC and DE. The MC/DE agreement is also excellent for the non-absorbing slab, and for large *ρ* values 〈*z*_*max*_|*ρ*〉 ≈ *s*_0_.

Finally, in Fig. 5e,f it is considered the total reflectance for the CW domain and 〈*z*_*max*_〉 and 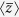 are reported as a function of *μ*_*a*_. These quantities are calculated both with DE, i.e. Eqs (28) and (31), and MC, for a semi-infinite medium (Fig. 5e) and for *s*_0_ = 20 mm (Fig. 5f). We have investigated a quite wide interval of *μ*_*a*_ ∈ [10^−4^, 1] mm^−1^. In general, for Fig. 5e,f, the agreement between DE solutions and MC results, although still good, is lower compared to that obtained for 〈*z*_*max*_|*t*〉, 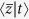, 〈*z*_*max*_|*ρ*〉 and 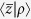. The reason of this fact is related to the deficiencies of the DE at short inter-optodes distances. For this reason, we have reported the DE solution (Eq. (28) for 〈*z*_*max*_〉) for two different positions of the source: 1) 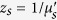 (the one adopted in the Theory section, solid curves)^42^,^43^,^44^; and 2) 
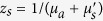
 (dotted curves) as adopted elsewhere^45^. We note that within the diffusion range (roughly up to *μ*_*a*_ = 0.1 mm^−1^) the two ways to account the source position are practically equivalent. Conversely, beyond the diffusion range, for very high value of *μ*_*a*_, the second choice shows a better agreement with the MC results. Thus, for high *μ*_*a*_ the choice of *z*_*s*_ becomes critical, because it strongly influences the solution of 〈*z*_*max*_〉_*DE*_ (Eq. (28)). In fact, the dependence of 〈*z*_*max*_〉_*DE*_ on *z*_*s*_ is mainly due to the breakdown of the DA. A real extension of this approach to very high *μ*_*a*_ values can only be indeed achieved by calculating 〈*z*_*max*_〉 with analytical solutions of the RTE; however, this is beyond the scope of this work. For *μ*_*a*_ = 1 mm^−1^ we note that the MC results for 〈*z*_*max*_〉 and 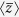 have about the same value close to zero. This is due to the fact that most of the detected trajectories for so large *μ*_*a*_ values have in average only about a single scattering event inside the slab. Under these physical conditions we have the failure of Eq. (31).

## Discussion

The results presented in the previous sections show the excellent reliability and accuracy of the analytical solutions based on the DE for calculating the penetration depth in diffusive media. The discrepancies observed between DE models and MC simulations are indeed not significant when the optical properties of the medium fall in the typical range of values of biological tissues and of many other diffusive media. The calculations are fast and all the algorithms can be easily implemented by most of the software packages largely used for scientific computing. This fact suggests that the penetration depth can become a ready-to-use information available to the investigators interested in modeling light transport in random media. This information achievable can be useful prior to perform experiments, in order to really probe the desired part of the medium investigated, or after the measurements have been carried out, in order to identify the actual depth of the medium probed by the detected light. The analytical formulae obtained for 〈*z*_*max*_|*t*〉_*DE*_ and 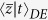 work indifferently well for any *μ*_*a*_ value. For the CW domain, the analytical formulae obtained for 〈*z*_*max*_|*ρ*〉_*DE*_, 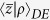, 〈*z*_*max*_〉_*DE*_ and 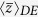 show to work surprisingly well for *μ*_*a*_ values up to 0.1 mm^−1^. Although we have shown all the results for 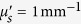, the scaling relationships originating from the similarity principle^42^, and valid for the solutions of the RTE, implies the validity of the model for other 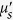 values.

The function 〈*z*_*max*_|*t*〉_*DE*_ (Eq. (17)) is fully characterized by the knowledge of the arrival time *t*, while the solution is independent of *ρ* and *μ*_*a*_. For a semi-infinite medium, for values of the optical properties typical of tissues, 〈*z*_*max*_|*t*〉_*DE*_ shows a time dependence given by a factor *t*^*α*^ with *α* ∈ [0.53, 0.57] and dependent on the refractive index mismatch between medium and external space (verified by fitting procedures, data not shown). These features make the study of the penetration depth in the TD particularly simplified as compared to the CW domain. It is curious to note that the *t* dependence of 〈*z*_*max*_|*t*〉_*DE*_ and 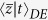 is not far from the *t* dependence of the average distance at which photons move away from an isotropic point source placed in an infinite medium after the emission time, and that within the DE is *t*^1/2^ (the calculation is straightforward by using the DE solution of ref. 42).

In the CW domain 〈*z*_*max*_|*ρ*〉_*DE*_ is strongly dependent on *μ*_*a*_ and *ρ* and shows a quite more complex dependence from the properties of the medium (Eq. (24)). In this domain 〈*z*_*max*_|*ρ*〉_*DE*_ can change dramatically also with small changes of *μ*_*a*_. For a non-absorbing semi-infinite medium 〈*z*_*max*_|*ρ*〉_*DE*_ depends linearly on *ρ* (Fig. 5c) as it is usually expected in the scientific community working in diffuse optics^39^. When absorption is added to the medium the dependence on *ρ* becomes proportional to *ρ*^*β*^ with *β* that is a function of *μ*_*a*_ and 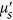 (verified by fitting procedures, data not shown). If *μ*_*a*_ = 0, then *β* = 1. When *μ*_*a*_ increases from zero, *β* reduces its values from 1 to values lower than 1. It must be noted that, for some ranges of *μ*_*a*_ and 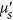 the dependence of 〈*z*_*max*_|*ρ*〉_*DE*_ from *ρ* can be approximated by *ρ*^2/3^ or by *ρ*^1/2^ as reported in refs 24,25. However, both these approximations do not have a general validity and only hold for these limited ranges of *μ*_*a*_ and 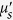. We have presented the validation of the analytical solutions of the penetration depth by using MC results obtained with scattering functions with asymmetry factor *g* = 0. Similar results were also obtained by using a scattering function with *g* = 0.9. We note that differences between the results obtained with *g* = 0 and *g* = 0.9 were appreciable only at early times and short source-detector distances where the diffusion conditions are not yet established.

The statistical approach proposed is general within the RTE (see Theory section). Indeed, by using proper solutions of the RTE it is possible to extend the validity of the presented analytical solutions beyond the diffusion approximation. We also stress that the DE solutions here presented are obtained modeling the pencil beam as an isotropic source placed at 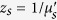. Thus, the slab thickness *s*_0_ has to be larger than *z*_*s*_ for obvious physical reasons.

Although we have presented analytical formulae of the penetration depth only for homogeneous geometries, the approach here proposed can be also applied, in all generality, to inhomogeneous media. In fact, the validity of Eqs (5), (6), (10) and (11), is not dependent on the considered geometry. In particular, making use of analytical solutions of the DE in layered geometries^46^,^47^,^48^,^49^,^50^, numerical evaluations of the relative 〈*z*_*max*_|*t*〉_*DE*_ and 〈*z*_*max*_|*ρ*〉_*DE*_ can be obtained with Eqs (5) and (10). Indeed, layered model geometries were employed in tissue optics, motivated by the fact that some tissues have a layered architecture. This is the case of muscle underneath a superficial fat layer or of the head with compartments such as scalp, skull, and brain^47^. A detailed analysis of this problem is out of the scope of this work, therefore we do not present any result in layered media. However, we must note that when dealing with a layered geometry some of the previous properties presented for the homogeneous case may be no longer exactly valid. In general 〈*z*_*max*_|*t*〉_*DE*_ may depend on the mismatch of absorption between the layers and on *ρ*. Thus, in layered media its time dependence may not be simply given by the factor *t*^*α*^ described above. It is also worth to stress that, in a layered medium where the optical properties are those typical of biological tissues, *R*_*DE*_(*ρ*, *t*) has a functional dependence on *ρ* similar to the homogeneous case^51^. Therefore, it can be shown that *f*_*DE*_(*z*|*t*) and 〈*z*_*max*_|*t*〉_*DE*_ are still approximatively independent of *ρ*.

In the past we have only few examples of analytical formulae of practical use allowing to obtain an estimation of the penetration depth. So far, we do not know of available validated formulae for the TD. For the sake of completeness, for CW domain, we remind the formulas provided by Zonios for 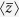 in a semi-infinite medium, and derived only from MC simulations. It is also worth to remind the approach based on the *δ*P-1 diffusion model with a *δ*-Eddington phase function proposed by Carp *et al.*^36^ in the CW domain and for a planar irradiation source to a semi-infinite medium. The same approach has been recently used in the frequency domain by Lee *et al.*^52^. As mentioned in the introduction, Carp *et al.*^36^ used a different metric for the definition of the penetration depth, but an expression of 〈*z*_*max*_〉 was not provided. Thus, from the previous literature dedicated to the penetration depth we can conclude that the set of analytical formulae here presented is the first that covers different domain of analysis and that has been validated for a wide range of optical and geometrical properties.

This study may have several potential uses. In tissue optics, near infrared spectroscopy or functional brain imaging, light is used to probe complex media and 〈*z*_*max*_〉_*DE*_ and 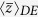 can help to define the part of tissue actually probed by the measurements. Moreover, the information led by 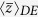 might be applicable to foresee some aspects of human vision when a diffusive medium is observed. In particular, 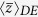 can provide an estimation of the thickness of the medium involved in the determination of the coloring vision perceived by our eyes. For example, in the research on painting materials it would be desired reducing the quantity of paint used to obtain a certain vision effect. The presented formulae can be of help in such investigations. In general, the penetration depth in diffusive media plays a key role in human vision since most of the natural media encountered in everyday-life are characterized by a diffusive regime of propagation. To this extent, it is interesting to note that, thanks to the reciprocity principle^53^,^54^, the DE solutions for *R*_*totDE*_(*z*) (Eq. (26)) are valid both for a pencil light beam and a plane wave source impinging onto the slab.

## Methods

To check the validity of the presented analytical formulae, we have modified a previously developed MC code^42^,^55^,^56^ to recalculate the same quantities without any approximation and by following a completely independent approach. For each detected trajectory *i* we have computed the mean value of the depth at which the photon undergoes scattering events, 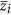, and the maximum value of the penetration depth, *z*_*max*,*i*_. For each temporal window of width Δ*t* at time *t*_*j*_ = *j*Δ*t*, we have stored the quantities


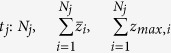


with *N*_*j*_ number of received photons inside the temporal windows at *t*_*j*_. Then, 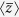 (*t*_*j*_), 〈*z*_*max*,*i*_〉 (*t*_*j*_) and *TPSF* (*t*_*j*_) were calculated as follow


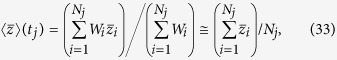



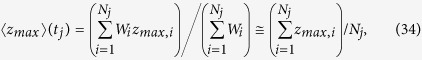



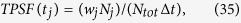


with *N*_*tot*_ the total number of launched photons and *w*_*j*_ the weight of the photons detected in Δ*t* at time *t*_*j*_ that is assumed for all photons equal to *w*_*j*_ = exp(−*μ*_*a*_*vt*_*j*_). In general, the weight factor *W*_*i*_ of each trajectory *i* that fall in the temporal window *j* may be different due to the width of the interval Δ*t*. We have selected an enough small Δ*t* so that we could assume for a given *j*: *W*_*i*_ ≈ *w*_*j*_, 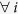 as done in Eqs (33, 34, 35).

From the MC simulations we also derived the corresponding values in the CW domain


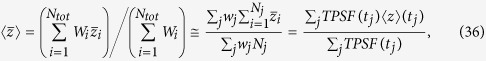



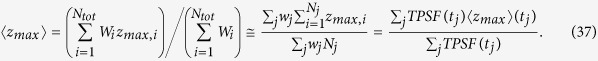


The calculation of the function *f*(*z*|*t*) has been implemented from the total reflectance. For each temporal window (we had 1000 windows) photons were classified inside an histogram (50 bins dividing the slab thickness) according to their *z*_*max*,*i*_ (bin width Δ*z*). The simulation provides us an integer matrix *M*_*j*,*k*_ that gives the number of detected trajectories in reflectance with a time of flight *t*_*j*_ and a *z*_*max*,*i*_ in the spatial window *k* at position *z*_*k*_. Therefore, we have that


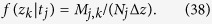


Similarly to *f*(*z*|*t*), we acted for the calculation of the function *f*(*z*|*ρ*) classifing for each detector the detected photons inside an histogram according to their *z*_*max*,*i*_. Following the above approach we have produced a set of MC results, with the Henyey-Greenstein model^42^ for the scattering function with the anisotropy factor *g* = 0 and *g* = 0.9, that we have compared to the analytical calculations obtained with the solutions of the DE.

## Conclusions

We have presented a comprehensive statistical approach describing the penetration depth in a random medium (infinite slab) in TD and CW domain. The presented theory is based on the concept of probability density functions that the photons emerging from the slab at a certain distance *ρ* and at time *t* have a maximum depth *z*_*max*_ in a given range. Analytical formulae of the probability density functions have been obtained within the framework of the DE. With the analytical solutions of the DE for the infinite slab we have derived analytical formulae for the mean maximum depth, 〈*z*_*max*_〉, and, with the help of an heuristic assumption, for the mean average depth, 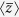, reached by the detected photons at the surface of the medium. By means of MC simulations we have also calculated the above functions within the RTE. Such results have been used as reference standard to check the validity of the analytical formulae obtained with the solutions of the DE. The DE formulae have shown to provide accurate predictions of the penetration depth, often also beyond the diffusive regime and therefore for all the typical values of optical properties in biological tissues and in other diffusive media.

## Additional Information

**How to cite this article**: Martelli, F. *et al.* There’s plenty of light at the bottom: statistics of photon penetration depth in random media. *Sci. Rep.* **6**, 27057; doi: 10.1038/srep27057 (2016).
